# Consensus care recommendations for alfapump^®^ in cirrhotic patients with refractory or recurrent ascites

**DOI:** 10.1186/s12876-022-02173-5

**Published:** 2022-03-08

**Authors:** Niels Kristian Aagaard, Massimo Malago, Andrea De Gottardi, Michael Thomas, Gerd Sauter, Cornelius Engelmann, David Aranovich, Michal Cohen, Thierry Thévenot, Thomas Ehmann, Jeroen Capel, Paolo Angeli, Rajiv Jalan, Guido Stirnimann

**Affiliations:** 1 Department of Hepatology and Gastroenterology, Aarhus University Hospital, Aarhus, Denmark; 2 Hepato-Pancreatic-Biliary and Liver Transplantation Surgery, Royal Free Hospital, University College London, London, UK; 3 Servizio di Gastroenterologia e Epatologia, Ente Ospedaliero Cantonale, Lugano, Switzerland; 4 Faculty of Biomedical Sciences, Università Della Svizzera Italiana, Lugano, Switzerland; 5 Department of General, Visceral, Tumour and Transplant Surgery, University Hospital of Cologne, Cologne, Germany; 6 Department of Internal Medicine II, LMU University Hospital, Munich, Germany; 7 Department of Hepatology and Gastroenterology, Charité – Universitätsmedizin Berlin, Berlin, Germany; 8 Hepatology and Bile Duct Surgery Service, Surgery B Department, Hillel Yaffe Medical Centre, Hadera, Israel; 9 The Liver Institute, Rabin Medical Centre, Beilinson Hospital, Petah Tikva, Israel; 10 Service d’Hépatologie, Hôpital Universitaire Jean Minjoz, Besançon, France; 11 Department of General and Visceral Surgery, St. Josef Krankenhaus Haan, Haan, Germany; 12 Sequana Medical NV, Zurich, Switzerland; 13 Facoltà di Medicina e Chirurgia, Università degli Studi di Padova, Padova, Italy; 14 Liver Failure Group, Institute for Liver and Digestive Health, Division of Medicine, UCL Medical School, Royal Free Campus, London, UK; 15 University Clinic for Visceral Surgery and Medicine, University Hospital Inselspital Bern and University of Bern, Freiburgstrasse 18, 3010 Bern, Switzerland

**Keywords:** Alfapump, Ascites, Cirrhosis, TIPSS, Refractory ascites, Paracentesis, Implantation, Long-term antibiotics, Patient management, Medical device

## Abstract

**Background:**

The alfapump^®^ is an implantable class III medical device that pumps ascitic fluid from the peritoneal space to the urinary bladder from where it is excreted. The pump reduces or abrogates the need for repeated paracentesis in patients with recurrent or refractory ascites.

**Aims:**

To improve outcomes for alfapump^®^ implantation and pre- and post-implant patient management in both clinical trial and real-world settings by development of consensus recommendations.

**Methods:**

The alfapump^®^ working group consisting of hepatologists and surgeons with extensive experience in implantation of the alfapump^®^ and patient management met on two occasions: (1) to determine the key areas where recommendations should be made; and (2) to discuss the experiences of the working group within those areas and formulate draft statements. Developed statements were submitted to the group and consensus sought on relevance and wording through a collaborative iterative approach in order to consolidate the recommendations into consensus statements. Only recommendations agreed upon unanimously were included.

**Results:**

Twenty-three consensus recommendations were developed in the areas of pre-implantation procedure, (three statements), surgical implant procedure (11 statements), immediate post-implant care (three statements) and long-term management (six statements).

**Conclusions:**

The consensus statements are a valuable reference resource for physicians managing patients with the alfapump^®^ and for those considering management strategies for patients with refractory ascites.

**Supplementary Information:**

The online version contains supplementary material available at 10.1186/s12876-022-02173-5.

## Introduction

Refractory ascites (RA) or recurrent ascites uncontrollable by diuretics and dietary measures are complications of advanced liver disease. If transjugular intrahepatic portosystemic shunt (TIPSS) is contraindicated, the primary management approach is repeated paracentesis [1, 2]. The alfapump^®^ (Sequana Medical NV, Ghent, Belgium), is an implantable medical device that moves ascites from the peritoneal space to the bladder, from where it is cleared by normal urination. The device has first received the CE-mark in 2011. It is currently available in all countries recognising this standard and in whose local language the instructions for use are available (Arabic, Danish, Dutch, English, French, German, Hebrew, Italian, Norwegian, Polish, Spanish, Swedish, Turkish). A pivotal clinical trial for market introduction is currently under way in the USA and Canada (POSEIDON; NCT03973866) and is expected to be completed in 2024. The device is fully reimbursed in Switzerland, under conditions for innovative treatments in Germany (NUB status 1) and under certain circumstances in Israel. In the UK, its use is recommended only with special arrangements regarding clinical governance, consent, audit or research [3].

Several studies, including a randomized controlled trial [4–7] and a meta-analysis [8], have proven the efficacy of the alfapump^®^ system in reducing paracentesis requirement and improvement of patient quality of life (QoL) [5, 7]. The device has also been evaluated as palliative treatment for malignant ascites [9]. Frequently occurring adverse events include device malfunction, acute kidney injury (AKI), spontaneous bacterial peritonitis (SBP) and urinary tract infection (UTI) [4–8]. In addition, increase of serum creatinine levels over time was observed [8, 10]. Still, providing appropriate care is maintained, safety can be well managed. The pump is wirelessly rechargeable through a hand-held charger that is also used to personalise pump programming and for device performance monitoring.

Development of the device continues, and introduction of a new peritoneal catheter [11] has substantially decreased related complications and re-intervention rate.

Optimal patient selection and monitoring, the development of a “recommended practice” implantation methodology, and refinement of post-procedural and long-term patient care are equally important to achieve optimal patient outcomes.

With this in mind, two expert meetings of European surgeons and hepatologists experienced in using the alfapump^®^ (referred to as alfapump^®^ user group in the following) were convened in October 2017 and February 2018 to share their experience, discuss and find consensus on standard care protocols, hereby generating recommendations for the use of the alfapump^®^ in patients with refractory or recurrent ascites. Recommendation statements updated with evidence generated in the meantime are included together with the corresponding rationales.

## Methodology

First, four primary areas were identified for which recommendations should be developed, corresponding to different phases of alfapump^®^ therapy: pre-implantation; implantation; post-implantation; and long-term follow-up.

By assessing the safety data from the PMSR [6], PIONEER [4] and RCT [5] studies, several points within each primary area were selected for further discussion, and draft definitions and recommendations were generated. The experimental protocols of all clinical studies were approved by the respective institutional review boards (IRBs) at the participating sites (for names and further details of IRBs/Ethics Committees, see Additional File 1: Tables S1, S2, and S3). Informed consent was obtained from all subjects and/or their legal guardian(s). All experimental protocols involving human data were in accordance to guidelines of national/international/institutional relevance and the Declaration of Helsinki. No new clinical data were generated for this manuscript, and there was no retrospective review of patient files outside of clinical studies. The datasets analysed during the current study are not publicly available as they are the property of Sequana Medical NV, but are available from Jeroen Capel (Jeroen.Capel@sequanamedical.com) on reasonable request.

Where applicable, other published literature was proposed as source of evidence and discussed. During the second meeting, the experts further reviewed the evidence and refined the points with robust arguments for or against incorporation in the recommendations. Discussion continued until consensus on statement wording was reached. If consensus was not found initially, experts’ concerns or queries were addressed by the group. Due to the limited availability of clinical data, no systematic standardised reporting system was applied. Only consensus statements agreed upon unanimously were included. Recommendation statements were updated with evidence generated in the meantime and approved by all authors.

## Results and discussion

### Pre-implantation: patient selection

The alfapump^®^ was developed for treatment of refractory ascites (RA) and “recurrent ascites”, a condition which does not fit the definition of RA but requires > 3 paracenteses per year despite diuretics and a low sodium diet [1, 2]. Its use is indicated mainly for RA and recurrent ascites with contraindications for insertion of a transjugular intrahepatic portosystemic shunt (TIPSS), including, but not limited to, advanced liver disease (model of end-stage liver disease [MELD] score ≥ 18/Child–Pugh class C), advanced age, cardiac/pulmonary/renal dysfunction, malignant disease, previous episodes of hepatic encephalopathy and bacterial infection [1–3]. A summary of indications and contraindications for alfapump^®^ treatment is provided in Table 1.

**Table 1:** Summary of indications and contra-indications

Indications	Relative contraindications	Contraindications
· RA due to liver cirrhosis with contraindications to TIPSS · Recurrent ascites due to liver cirrhosis that is poorly controlled by diuretics and dietary measures (> 3 paracenteses per year) and contraindications to TIPSS	· Advanced sarcopenia · Bed confinement · Hepatorenal syndrome · Contraindications to anaesthesia · Significant peripheral oedema	· Ongoing SBP · Abdominal skin infections · Loculated ascites · Obstructive uropathy

Nutritional status and frailty of the patient are important, and it is pointed out that a computed tomography (CT) scan to assess sarcopenia can be performed as for guidance in the decision-making process.

Patients with significant peripheral oedema are difficult to manage due to pre-existing fluid balance issues. In case of ongoing SBP, a stand down period of one week following successful treatment is recommended. A urologist should assess prostate-related obstructive uropathy in men. Patients who exhibit septated or loculated ascites and untreated obstructive uropathy should be definitively excluded. Sarcopenic patients who are bedbound (frailty score 4 [1]), those with established hepatorenal syndrome and those unsuitable for anaesthesia should not be definitively excluded but extreme caution is advised.

Patients should be managed by a multidisciplinary team (MDT), similar to recommendations for elective hernia repair in patients with cirrhosis [2]. In addition to hepatologist, surgeon, anaesthetist and nursing staff, inclusion of a dietician in the MDT should be considered. As nutritional status influences outcome, identified deficits should be managed. If the patient is awaiting liver transplantation, the transplant surgeon should be involved and, in case of men with prostate-related obstructive uropathy, a urologist should be consulted. Finally, it should be established if the patient has adequate home care. All staff involved in patient management (nursing, intensive care unit [ICU], emergency room and home care staff) should be aware of the pump and receive appropriate information and education.

In patients pre-selected for alfapump^®^ implantation the use of non-selective beta-blockers (NSBB) should be reviewed based on the 2018 European Association for the Study of the Liver (EASL) recommendations in the context of variceal bleeding prophylaxis [1]. The 2021 American Association for the Study of Liver Diseases (AASLD) guidance [2] states that there is a controversy but, similar to the recent British guideline [3], does not necessarily see contraindications in patients with RA. All guidelines agree that in patients with RA and hypotension [2] as indicated by a systolic blood pressure < 90 mmHg [1, 2], or hyponatremia with serum natremia < 130 mmol/L, or serum creatinine > 1.5 mg/dL [2] indicating deterioration of glomerular filtration rate, the NSBB dose should be reduced or even temporarily discontinued. The EASL guidelines specifically discourage the use of carvedilol and advise discontinuation in case of acute events such as bleeding, sepsis, SBP or AKI [1]. More data are required before more detailed recommendations can be made.

#### Recommendations

1. *“Patients with liver cirrhosis and refractory or difficult-to-treat ascites should be assessed for suitability for alfapump* ^®^ *treatment if treatment by TIPSS is contraindicated. Patients with loculated ascites, untreated obstructive uropathy and SBP should be excluded. Sarcopenic, bedridden patients, those with hepatorenal syndrome (HRS) and those unsuitable for anaesthesia can be treated, but with extreme caution. Concomitant repair of hernias is discouraged and extra care should be taken with peripheral oedema patients.”*
2. *“All medical professionals involved in treatment and management of the patient, including, but not limited to, hepatologist, surgeon, dietician, nurses, anaesthetist, ICU staff, urologist, and home caregiver should be consulted. Care decisions should be made at the pre-surgical phase.”*
3. *“The use of non-selective beta-blockers should be reviewed in every patient pre-selected for alfapump* ^®^ *implantation based on current guidelines.”*

### Implantation procedure

This phase covers patient admission and preparation procedures, correct implantation technique, including bladder catheter installation, peritoneal catheter positioning and suturing, pump pocket location, system testing, and complication resolution.

The pump has been successfully implanted using both open surgery and interventional radiology [12, 13].

The patient is admitted up to 48 h prior to implant to perform general laboratory tests and to exclude SBP by ascites culturing and determination of neutrophil count. Diuretics should be discontinued to minimise the risk of AKI. The surgeon should examine the patient for signs of skin infection and determine the preferred site for pump pocket location, which may be influenced by scarring or other physiological features. Pre-implant paracentesis should be performed, but care should be taken to leave sufficient ascitic fluid volume for device testing in the operating room. If the patient is admitted less than 48 h prior to surgery, the MDT should ascertain that the above-mentioned procedures are correctly performed.

Prophylactic antibiotic use (e.g., 400 mg norfloxacin qd if available [1, 2], 500 mg oral ciprofloxacin qd [2], or equivalent as per local guidelines, taking into account local resistance patterns) is encouraged to reduce the incidence of pre-implantation infections. Patients with cirrhosis have impaired immune function and tend to perform poorly with inadequate antibiotic prophylaxis. Antibiotics should not be discontinued while the alfapump^®^ is implanted and not without consultation of the treating hepatologist.

Colonisation of the pump may result in untreatable infection. Accordingly, patients should be monitored carefully for post-procedural infections, especially those with previous infections (SBP, UTI, multi-drug resistant [MDR] infections) and diabetes. As cirrhotic patients frequently exhibit poor wound healing, sutures should not be removed earlier than two to three weeks post-surgery to minimise risk of wound dehiscence.

Detailed descriptions of implantation procedures were published elsewhere [12, 13]. Critical considerations are highlighted in the consensus recommendations.

#### Recommendations

(4) *“The patient may be admitted up to 48 h pre-implant. Diuretics should be discontinued. Antibiotic prophylaxis should be initiated at admission and paracentesis performed to ensure a flat abdomen and to exclude SBP. Sufficient fluid should be left for in-surgery device testing. Surgeons should personally examine the patient to determine implant site.”*
(5) *“Consider initiating prophylactic antibiotic treatment (norfloxacin 400 mg qd, oral ciprofloxacin 500 mg qd or equivalent) 48 h prior to implant in hospitalised patients and at admission in patients admitted less than 48 h before surgery. Prophylaxis should ideally be continued from admission (i.e., pre-implantation) to alfapump* ^®^ *explant. Intravenous antibiotics should be used from 30 min prior to surgery but not exceeding 48 h post-surgery.”*
(6) *“Alfapump* ^®^ *implantation should be performed only by highly experienced and trained surgeons. All operating room (OR) staff should be familiar with the device and a Sequana Medical implant professional should assist.”*
(7) *”To prevent wound dehiscence or infection, the patient should take an antiseptic shower prior to surgery and current (dermal) infections should be ruled out. Precise clean incisions, bleeding control, and adequate pump pocket sizing to avoid tension are encouraged (use single sutures if necessary). Sutures should remain in place for two to three weeks but drains should be removed as soon as possible.”*
(8) *“The pump pocket should be positioned two to four cm below the costal margin, with enough room to house the pump comfortably in different postures. Distance to the peritoneal incision site should be maximised. The pump should be fixed with two non-resorbable stiches, and the pocket should be closed in layers with tension-free sutures; kinking or rotation of the catheters should be avoided. Consider positioning below the fascia in emaciated patients.”*
(9) *“Puncture of the bladder should be strictly suprapubical and extraperitoneal. The use of ultrasound ensures correct localisation and puncture direction. The bladder should not be drained until the catheter is inserted. The bladder catheter felt cuff should be fixed to the subcutaneous tissue or subcutaneous fascia with non-resorbable stiches. Dyed fluid runout should be checked before closure.”*
(10) *“The peritoneal catheter should be fixated with non-resorbable water tight purse-string-suture at the peritoneum, with the felt cuff inside the rectus muscle and the silicon ball inside the peritoneal cavity.”*
(11) *“Correct catheter length is important to avoid tension if too short, or kinking if too long—the length can only be judged correctly when the abdomen is flat.”*
(12) *“The pump should be tested in the OR prior to closing, with an initial pumping rate set to 120% of the expected daily volume.”*
(13) *“Keeping ascites accumulation minimal and abdominal pressure low during and especially after surgery is crucial to reduce the potential for dislocated catheters, peritoneal leakage, pump pocket fluid collection and wound dehiscence.”*
(14) *“The Smart Charger should leave the OR together with the patient.”*

### Post-implantation care

This section covers the immediate surgical recovery period with recommendations on pump settings, monitoring, management of acute procedure-related complications, wound healing, suture removal and discharge.

Use of non-steroidal anti-inflammatory drugs (NSAIDs), angiotensin-converting enzyme inhibitors, angiotensin II antagonists, a1-adrenergic receptor blockers or aminoglycosides should be avoided in all cirrhotic patients [1].

Alfapump^®^ total daily volume (TDV) should be kept < 1 L whenever possible. Dietary measures to reduce ascites production should be implemented if necessary.

Minimising intra-abdominal pressure prevents tension on sutures and fluid leakage into the pump pocket and thus supports wound healing. This requirement has to be balanced with avoiding AKI in the early post-operative period. Both continuous fluid drainage and intra-abdominal pressure from tense ascites may lead to AKI. Nonetheless, especially during the surgical recovery period until wounds are healed, priority should be given to minimising abdominal pressure as AKI may be managed through appropriate measures as per current guidelines [1, 2].

There are insufficient data to recommend any albumin substitution level, amount or frequency suitable for all patients [1, 2]. Consequently, albumin should be substituted as per local standards. Serum creatinine ≥ 0.3 mg/dl above pre implant level or 1.5–twofold increase thereof indicates stage 1 AKI, which should be diagnosed and treated according to current guidelines [1, 2]. Regular substitution should be considered in patients with TDV > 1 L.

#### Recommendations

(15) *“Avoid NSAIDs, angiotensin-converting enzyme inhibitors, angiotensin II antagonists, a1-adrenergic receptor blockers or aminoglycosides.”*
(16) *“The post-surgical TDV should be sufficient to maintain a flat abdomen but should ideally not exceed 1 L. If AKI develops, treat as per guidelines with appropriate albumin infusion  with or without terlipressin.”*
(17) *“Albumin should be substituted per current guidelines or institutional guidelines of the expert centre as required. Consider regular substitution in patients with TDV* > *1 L.”*

### Long term care

This section covers patient and caregiver training and support, long-term prophylaxis, monitoring, nutrition and long-term complications.

Patients’ health care is often managed by general practitioners (GPs). Alfapump^®^-specific treatment should not be changed without consulting the hepatologist. The GP and other health care providers, especially those whose assessments and/or treatments could potentially influence pump function, should be made aware of limitations (such as, importantly, device damage by magnetic resonance imaging [MRI] and long-term complications) to facilitate early identification and appropriate referral.

Patients and their primary caregiver should be familiarised with pump use, especially charging, diet guidance and use of over-the-counter analgetics. Importantly, they should be familiar with early signs of complications and should be encouraged to contact the hepatologist or implant centre upon detection of any warning signs. Educational material should be provided by the centre and the patient should be walked through the important points before discharge.

Dietary restrictions may be relaxed in order to improve quality of life, general nutrition and sarcopenia. Guidelines [14] may be a useful starting point, however, adherence to a strict low sodium diet might be difficult. A protein-rich “no added salt” diet is proposed. In case of TDV increase to volumes larger than 1 L following dietary changes, a sodium-restricted diet needs to be re-introduced.

Fluid intake should not exceed 2 L per day. Hyponatremia should be managed as in any other cirrhotic patient (fluid intake restriction) and the pump settings left unaltered. Excessive fluid intake might result in ascites volumes that are uncontrollable by the pump.

Similar to post-implantation care, albumin should be substituted according to current guidelines [1, 2, 15] and local standards of the treating expert centre.

An overview of symptoms and management of common complications that may appear in the long term is provided in Table 2. Although the risk of developing MDR infections related to long-term antibiotic prophylaxis remains a matter of concern [16], patients receiving alfapump^®^ are at a very high risk of infection, and consequently antibiotic prophylaxis should be continued until explant.

**Table 2:** Symptoms and management of long-term complications

Clinical symptom	Diagnosis/complication	Management
Distended abdomen	Ascites accumulation (Device malfunction)	Paracentesis Referral to expert centre Determination of type of device malfunction Surgical re-intervention if indicated
Elevated protein in urine	Ascites in urine (consequence of alfapump^®^ action)	No reason for concern if no other signs of UTI are present
Fever (≥ 2 days)	Infection · SBP · Device colonisation · UTI	Initiation of antibiotic treatment Referral to expert centre Diagnostic paracentesis for exclusion of SBP Identification of causative agent incl. antibiotic susceptibility profile in collaboration with local infection management Explant pump if no signs of improvement after initiation of antibiotic treatment
Implant site redness, swelling, or pain	Cellulitis Ascites leakage into pump pocket	Initiate antibiotic treatment Revision of device (catheter dislocation?) Surgical re-intervention if indicated
Increase of serum creatinine ≥ 0.3 mg/dl or 1.5–twofold above pre-implant levels	AKI	Treatment with albumin infusion with or without terlipressin according to guidelines and local standards (Temporary) reduction of TDV
Urination problems	Dehydration AKI	Revision of fluid management (Temporary) reduction of TDV Treatment with antibiotics, terlipressin and albumin infusion according to guidelines and local standards
Wound leakage	Wound dehiscence Catheter dislocation (ascites leakage into pump pocket)	Wound revision Surgical re-intervention if indicated

Successful antibiotic treatment of bacterial colonisation of the pump or catheters is possible, but explant may be indicated. However, it should be considered that any surgical intervention, including for pump removal, also carries a substantial risk in this patient type. Decisions on management of such infections should be guided by a risk–benefit analysis for each individual patient and follow institutional standards. Patients should receive a discharge letter advising them to immediately contact their hepatologist in case of wound leakage, implant site redness, swelling or pain, ascites accumulation, fever for > 2 days, urination problems or suspicion of a bladder infection.

It is encouraged to promptly initiate antibiotic treatment in case of infection and, in parallel, consult local infection management to identify the causative agent and determine its antibiotic susceptibility profile, whenever feasible and even if the decision is taken to explant the device. This approach will accelerate appropriate treatment in case of MDR infection [2].

If a patient dies with the alfapump^®^, device removal is mandatory (except in Israel).

#### Recommendations

(18) *“System or implant site infection may be successfully treated with antibiotics, or the system may be explanted. The decision to explant should be made on a case-by-case basis. Patients should be provided with a discharge letter advising them of signs and symptoms that may indicate infection or other serious complications.”*
(19) *“The patient and caregiver should be trained using all available material. The GP should receive a discharge letter detailing all important aspects of alfapump* ^®^ *treatment (ascites-related proteinuria, referral to the expert centre in case of infection, ascites build-up, reduced kidney function, wound healing issues or intended changes in antibiotic regimen, no MRI examinations).”*
(20) *“Patients should maintain a protein-rich normal diet avoiding high-salt foods and aim for moderate fluid intake.”*
(21) *“Albumin should be substituted per current guidelines or institutional guidelines of the expert centre.”*
(22) *“Antibiotic prophylaxis should continue for the duration of alfapump* ^®^ *treatment.”*
(23) *“Device removal after death is mandatory (except in Israel)”.*

## Conclusions

These recommendations are intended to formalise the procedures and care that achieve optimal outcomes for RA patients treated with the alfapump^®^. Further clinical studies will provide answers to outstanding questions, especially regarding patient selection and long-term alfapump^®^ therapy.

## Supplementary Information

**Additional file 1: Tables**. Lists of IRBs/Ethics Committees that approved the experimental protocols of the clinical trials the consensus recommendations are based on.

## Data Availability

The datasets analysed during the current study are not publicly available as they are the property of Sequana Medical NV, but are available from Jeroen Capel (Jeroen.Capel@sequanamedical.com) on reasonable request.

## Abbreviations

AKI: Acute kidney injury
AASLD: American Association for the Study of Liver Diseases
CT: Computed tomography
EASL: European Association for the Study of the Liver
GP: General practitioner
HRS: Hepatorenal syndrome
ICU: Intensive care unit
IRB: Institutional Review Board
MDR: Multi-drug resistant
MDT: Multidisciplinary team
MRI: Magnetic resonance imaging
NSAID: Non-steroidal anti-inflammatory drug
NSBB: Non-selective beta-blocker
NUB: Neue Untersuchung oder Behandlung (German; English: Innovative Examination or Treatment)
OR: Operating room
qd: Every day
RA: Refractory ascites
SBP: Spontaneous bacterial peritonitis
TDV: Alfapump^®^ total daily volume
TIPSS: Transjugular intrahepatic portosystemic shunt
UTI: Urinary tract infection
