# Supplementary material for: Consensus care recommendations for alfapump® in cirrhotic patients with refractory or recurrent ascites
Source: BMC Gastroenterol. 2022 Mar 8;22:111. doi: 10.1186/s12876-022-02173-5 (PMC8905806; doi:10.1186/s12876-022-02173-5)
Supplement: Supplementary file 1 — Additional file 1: Tables. Lists of IRBs/Ethics Committees that approved the experimental protocols of the clinical trials the consensus recommendations are based on. [file 12876_2022_2173_MOESM1_ESM.docx]

# Supplementary Tables

Consensus Care Recommendations for alfapump^®^ in Cirrhotic Patients with Refractory or Recurrent Ascites

**Niels Kristian Aagaard, MD, PhD^1^; Massimo Malago, MD, PhD^2^; Andrea De Gottardi, MD, PhD^3^; Michael Thomas, MD^4^; Gerd Sauter, MD^5^; Cornelius Engelmann, MD^6^, David Aranovich^7^, MD; Michal Cohen, MD^8^; Thierry Thévenot,MD^9^, Thomas Ehmann, MD^10^, Jeroen Capel, MSc^11^, Paolo Angeli, MD, PhD^12^; Rajiv Jalan, MBBS, MD, PhD, FRCPE, FRCP^13^, Guido Stirnimann. MD^14^**

Table S 1: List of Institutional Review Boards and Ethics Committees that approved the experimental protocol of the alfapump^®^ Post-Marketing Surveillance Registry (PMSR; NCT1532427) (1).

| **Institutional Review Board/  Ethics Committee** | **Address** | **Date of Approval** |
| --- | --- | --- |
| Kantonale Ethikkommission Bern KEK | Postfach 56, 3010 Bern, SWITZERLAND | 26.Feb.15 |
| Ärztekammer des Saarlandes | Faktoreistrasse 4,  66111 Saarbrücken, GERMANY | 19.Mar.12 |
| Ethik-Kommission an der Medizinischen Fakultät der Universität Leipzig | Käthe-Kollwitz-Strasse 82,  04109 Leipzig, GERMANY | 20.Nov.12 |
| Ethikkomission Universitätsklinikum Frankfurt | Theodor-Stern-Kai 7, 60596 Frankfurt, GERMANY | 27.Sep.12 |
| Ethik-Kommission bei der Medizinischen Fakultät der Universität Würzburg | Versbacherstrasse 9, 97078 Würzburg, GERMANY | 24.May.13 |
| Ethik-Kommission Universitätsklinikum Jena | Bachstrasse 18, 07743 Jena, GERMANY | 16.Jul.14 |
| Ethikkommission an der Technischen Universität Dresden | Fetscherstrasse 71, 01307 Dresden, GERMANY | 11.Mar.14 |
| Sächsische Landesärztekammer | Postfach 100465, 01074 Dresden, GERMANY | 07.Jul.14 |
| Ärztekammer Nordheim | Tersteegenstrasse 9, 40474 Düsseldorf, GERMANY | 15.May.15 |
| Comité Ético de Investigación Clínica y Comisión de Proyectios de Investigación del Hospital Unversitari Vall d’Hebron | Pg. Vall d’Hebron,  119-129 08035 Barcelona, SPAIN | 09.Oct.12 |

| **Institutional Review Board/  Ethics Committee** | **Address** | **Date of Approval** |
| --- | --- | --- |
| NRES Committees - North of Scotland | Summerfield House 2 Eday Road Aberdeen AB156RE, UNITED KINGDOM | 21.Feb.13 |

Table S 2: List of Institutional Review Boards and Ethics Committees that approved the experimental protocol of the PIONEER study (NCT01030185) (2).

| **Institutional Review Board/  Ethics Committee** | **Address** | **Date of Approval** |
| --- | --- | --- |
| Agencia Valenciana de Salut (Departamento de Salut 19) | Avenida Pintor Baeza, 12 - 03010 – Alicante, SPAIN | 30.Jun.09 |
| Secretaria del Comité Ético de Investigación Clínica de La Fundació de Gestió Sanitaria del Hospital de la Santa Creu I Sant Pau de Barcelona | Sant Antoni Ma Claret, 167 - 08025 Barcelona, SPAIN | 29.Sep.10 |
| Ethikkomission des Fachbereichs Medizin der J. W. Goethe-Universität Frankfurt am Main | Strassenbahn 12, 15, 21 - 60590 – Frankfurt, GERMANY | 20.Jul.09 |
| National Research Ethics Service, Outer North London REC | Watford Road - Harrow - HA13UJ – Middlesex, UNITED KINGDOM | 15.Mar.10 |
| Ethikkomission an der Universität Regensburg | Franz-Josef-Strauss-Allee 11 - 93053 – Regensburg, GERMANY | 21.Jan.10 |
| Ethikkomission der Medizinischen Fakultät, Rheinische Friedrich - Willhelms Universität Bonn | Sigmund-Freud-Strasse 25 - 53105 – Bonn, GERMANY | 26.Apr.10 |
| Commissie Medische Ethiek van der Universitaire Ziekenhuizen Kuleuven | Herestraat 49 - B3000 – Leuven, BELGIUM | 18.Oct.10 |
| ETHICS COMMISSION FOR MULTICENTER TRIALS | 8 Damyan Gruev Str., 1303 - Sofia - BULGARIA | 10.Dez.10 |

Table S 3: List of Institutional Review Boards and Ethics Committees that approved the experimental protocol of the alfapump® randomized clinical trial (RCT; NCT01528410) (3, 4).

| **Institutional Review Board/  Ethics Committee** | **Address** | **Date of Approval** |
| --- | --- | --- |
| NREC East of England (Cambridge East) | Victoria House - Fulbourn - Cambridge CB21, UNITED KINGDOM | 29.Mar.12 |
| Comité de Protection des Personnes Ile de France IV | 1 avenue Claude Vellefaux 75475 – Paris, FRANCE | 03.Jan.13 |
| Ethikkomission Medizinische Universität Wien | Borschkegasse8b/6 - 1090 – Wien, AUSTRIA | 09.Oct.12 |
| Comité Ético de Investigación del Hospital Universitari Vall d´Hebron | Pg. Vall d´Hebron, 119 - 129 - 08035 – Barcelona,SPAIN | 11.Jul.12 |
| Comitato Etico della Provincia di Padova | Via Giustiniani, 1 - 35128 – Padova, ITALY | 02.Mar.15 |
| NHS Foundation Trust - Research and Innovation University Hospitals Bristol NHS Foundation Trust | Upper Maudlin Street Bristol BS2 8AE, UNITED KINGDOM | 03.Jan.13 |

**References**

1. Stirnimann G, Berg T, Spahr L, Zeuzem S, McPherson S, Lammert F, et al. Treatment of refractory ascites with an automated low-flow ascites pump in patients with cirrhosis. Alimentary pharmacology & therapeutics. 2017;46(10):981-91.

2. Bellot P, Welker MW, Soriano G, von Schaewen M, Appenrodt B, Wiest R, et al. Automated low flow pump system for the treatment of refractory ascites: a multi-center safety and efficacy study. J Hepatol. 2013;58(5):922-7.

3. Bureau C, Adebayo D, Chalret de Rieu M, Elkrief L, Valla D, Peck-Radosavljevic M, et al. Alfapump(R) system vs. large volume paracentesis for refractory ascites: A multicenter randomized controlled study. J Hepatol. 2017;67(5):940-9.

4. Stepanova M, Nader F, Bureau C, Adebayo D, Elkrief L, Valla D, et al. Patients with refractory ascites treated with alfapump(R) system have better health-related quality of life as compared to those treated with large volume paracentesis: the results of a multicenter randomized controlled study. Quality of life research : an international journal of quality of life aspects of treatment, care and rehabilitation. 2018;27(6):1513-20.
